# A promising anti-tumor targeting on ERMMDs mediated abnormal lipid metabolism in tumor cells

**DOI:** 10.1038/s41419-024-06956-4

**Published:** 2024-08-04

**Authors:** Mingshi Pang, Liuchunyang Yu, Xiaoyu Li, Cheng Lu, Cheng Xiao, Yuanyan Liu

**Affiliations:** 1 School of Chinese Materia Medica, Beijing University of Chinese Medicine, Beijing, China; 2 Institute of Basic Research in Clinical Medicine, China Academy of Chinese Medical Sciences, Beijing, China; 3 Institute of Clinical Medicine, China-Japan Friendship Hospital, Beijing, China

**Keywords:** Cancer metabolism, Cancer therapy

## Abstract

The investigation of aberrations in lipid metabolism within tumor has become a burgeoning field of study that has garnered significant attention in recent years. Lipids can serve as a potent source of highly energetic fuel to support the rapid growth of neoplasia, in where the ER-mitochondrial membrane domains (ERMMDs) provide an interactive network for facilitating communication between ER and mitochondria as well as their intermembrane space and adjunctive proteins. In this review, we discuss fatty acids (FAs) anabolic and catabolic metabolism, as well as how CPT1A-VDAC-ACSL clusters on ERMMDs participate in FAs transport, with a major focus on ERMMDs mediated collaborative loop of FAO, Ca^2+^ transmission in TCA cycle and OXPHOS process. Here, we present a comprehensive perspective on the regulation of aberrant lipid metabolism through ERMMDs conducted tumor physiology might be a promising and potential target for tumor starvation therapy.

## Facts

- Lipids can serve as a potent source of highly energetic fuel to support the rapid growth of neoplasia.
- The ERMMDs as important membrane systems coordinate the function of ER and mitochondria in lipid anabolic and catabolic metabolism.
- The ERMMDs participate in lipid metabolism through substance transportation, signaling transmission and functional coordination.
- The regulation of aberrant lipid metabolism through ERMMDs conducted tumor physiology might be a promising and potential target for tumor starvation therapy.

## Questions

- How does the interaction between mitochondria and ER with the connected membrane systems maintain cell biological stability?
- How the exact mechanism of ERMMDs does mediated collaborative loop work between ER and mitochondria?
- What other potential targets located on ERMMDs can provide ideas for cancer treatment?

## Introduction

Cancer is essentially a disorder in cellular proliferation and metabolism, and the disrupted metabolism of cancer cells often leads to the accumulations of metabolic intermediate products, which can serve as sources for other substances [1]. The metabolism of glucose serves as the predominant energy source of normal cells, which are enhanced in cancer cellular uptake is alternatively directed towards anabolic pathways, including protein glycosylation and ribosylation, rather than being used for adenosine triphosphate (ATP) production through tricarboxylic acid (TCA) cycle [2]. Consequently, the significance of lipids as an essential supplier of high-energy fuel in cancer proliferation cannot be overlooked. The energy homeostasis in cancer cells is maintained by fatty acids (FAs) through both anabolic and catabolic metabolism. The presence of higher de novo lipogenesis (DNL) in cancer cells results in the accumulation of synthesized FAs as neutral lipid triglycerides (TAG) within the bilayers of endoplasmic reticulum (ER) membranes, ultimately resulting in the formation of lipid droplets (LDs) [3]. Upon tumor starvation stress, the required free FAs are derived not only from the extracellular environment but also from DNL, especially from TAG stored in LDs [4]. After translocating into the mitochondria, FAs undergo β-oxidation to generate acetyl-CoA, which subsequently enters the TCA cycle to produce nicotinamide adenine dinucleotide (NADH) and flavin adenine dinucleotide (FADH_2_). The reducing equivalents transfer electrons to the inner mitochondrial membrane (IMM) oxidative respiratory chain, thereby generating ATP for rapid tumor cells proliferation [5].

Importantly, the transportation of FAs depends on the extensive ER membranous network, which spans throughout the cellular interior and establishes stable connections with that of inner and outer mitochondria membranes as well as the contacts between the membranes, referred to as ER-mitochondrial membrane domains (ERMMDs). Physical contacts known as ERMMDs create a unique protein-protein interaction environment that regulate lipid biosynthesis and metabolism, in addition to other crucial processes such as mediating signal transduction and establishing an interactive network between the two organelles [6, 7]. For instance, the existence of a specific and functional protein cluster, known as CPT1A-VDAC-ACSL clusters, on ERMMDs may facilitate the direct translocation of FAs into the intermembrane space (IMS) [8]. Furthermore, Ca^2+^ stored in the ER can be transported through the IP3R-GRP75-VDAC protein on ERMMDs into mitochondria and regulate its production of reactive oxygen species (ROS), thereby regulate lipid metabolism by activating enzymes associated in the TCA cycle [9]. Thus, the collaborative functional loop between ER and mitochondria relies on the interaction of ERMMDs, which plays a pivotal role in maintaining abnormal lipid metabolism of cancer cells, ultimately influencing cancer progression.

In this review, we explore the metabolic processes of cancer cells, focusing on the pathways involved in FAs synthesis and metabolism. In particular, we emphasize that physical associations between CPT1A-VDAC-ACSL clusters can directly transport FAs into the IMS. Throughout, we emphasize the emerging role of ERMMDs in coordinating the function of two organelles as ER and mitochondria, including their involvement in substance transportation, signaling transmission and functional coordination.

## FAs act as high-energy fuel in tumor pathophysiology

Cancer-associated alterations in lipid metabolism appear to be specific to each tumor type, enabling the cells to meet their energetic demands for survival and rapid proliferation of tumor cells. The abnormal lipid metabolism provides FAs as high-energy fuel, allowing some cancers to up-regulated for fatty acids oxidation (FAO), while others exhibit a greater dependence on lipid synthesis [10]. Here, detailed dissection of the sequential events on how DNL occurs in the ER and is ultimately stored in LDs is summarized to provide an understanding of this dysfunctional process. While, the way of LDs is catabolized by lipolysis and lipophagy to produce free FAs for cellular use are discussed in detail.

### Alterations of energy metabolism in neoplasia

The metabolic demands of tumor cells far surpass those of other tissues, and cancer cells adeptly adjust their energy metabolism to meet the heightened requirements for growth and proliferation [11]. Metabolic alterations are as follows: (i) Even in the presence of oxygen, cancer cells exhibit a predilection for metabolizing glucose through glycolysis, an intriguing phenomenon commonly referred to as the Warburg effect. The complete oxidation of a glucose molecule yields an impressive 36 ATP molecules, whereas glycolysis generates only 2 ATP molecules [1]. Besides, this seemingly wasteful aerobic glycolysis process also provides carbon precursors and importantly intermediates for the synthesis of nucleic acids, proteins, FAs and other molecules [12]. (ii) The metabolism of glutamine is upregulated in cancer cells. Glutamine can undergo metabolic process to produce α-ketoglutarate, which subsequently passes into the TCA cycle and can be converted back to citric acid. Citric acid, in turn, serves as a precursor for FAs production [13]. (iii) The dysregulation of lipid metabolism, characterized by enhanced DNL and increased utilization of lipids as an energy source, represents prominent metabolic abnormalities observed in tumor cells. The role of lipids extends beyond the mere formation biological membranes; they also serve as reservoirs for storing energy and signaling molecules [14]. The Warburg effect has significantly influenced metabolic studies on cancer. Today, the current consensus suggests that most cancer cells predominantly depend on mitochondrial metabolism, utilizing substantial quantities of pyruvate derived from FAs for ATP production via oxidative phosphorylation (OXPHOS) [1, 15]. Comparatively speaking, in term of their per unit mass, FAs yield twice as much ATP as carbohydrates, while stored FAs like TAG provide six times as much ATP as glycogen [2]. The FAs, acting as a potent source of high-energy fuel, play a pivotal role in supplying ATP to cancer cells, thereby facilitating the rapid proliferation of tumor cells.

### DNL and storage of FAs

The FAs can be absorbed either through direct uptake from the plasma or via DNL, a metabolic pathway responsible for synthesizing FAs using surplus carbon sources like glucose and amino acids [16]. The DNL process initiates with palmitate synthesis in the intermembrane space between ER and OMM. Subsequently, palmitate permeates into the two leaflets of the ER membrane to generate FAs that undergo modifications by various elongases and desaturases [17]. Then, FAs undergo a series of enzymatic reactions to generate neutral lipids, such as TAG, which accumulate within the leaflets of the ER membrane and subsequently give rise to LDs-specialized in the storage of lipids [18].

In normal conditions, DNL is actively engaged in the primary metabolic tissues including adipose tissue and liver; however, a multitude of DNL also exists within cancer cells ever in the presence of exogenous uptake of FAs [19]. The intracellular pool of FAs, derived from both DNL and exogenous uptake, serves as the foundation for synthesizing TAG for energy storage, as well as cardiolipin (CL), glycerophospholipids and sphingolipids for membrane synthesis [18]. In cancer cells, reduced FAs synthesis during hypoxia may be compensated by an augmented uptake of external lipids. For instance, an investigation of the effects of FAs on hypoxic cancer cells revealed that hypoxia resulted in an enhanced uptake of lipids, particularly monounsaturated acyl chains [20]. A separate study revealed that hypoxia-induced periplasmic lipoprotein 2 (PLIN2) promotes lipid storage in LDs, which subsequently serves as a reservoir for energy production and antioxidant defense upon re-oxygenation [21].

**Fig. 1:**
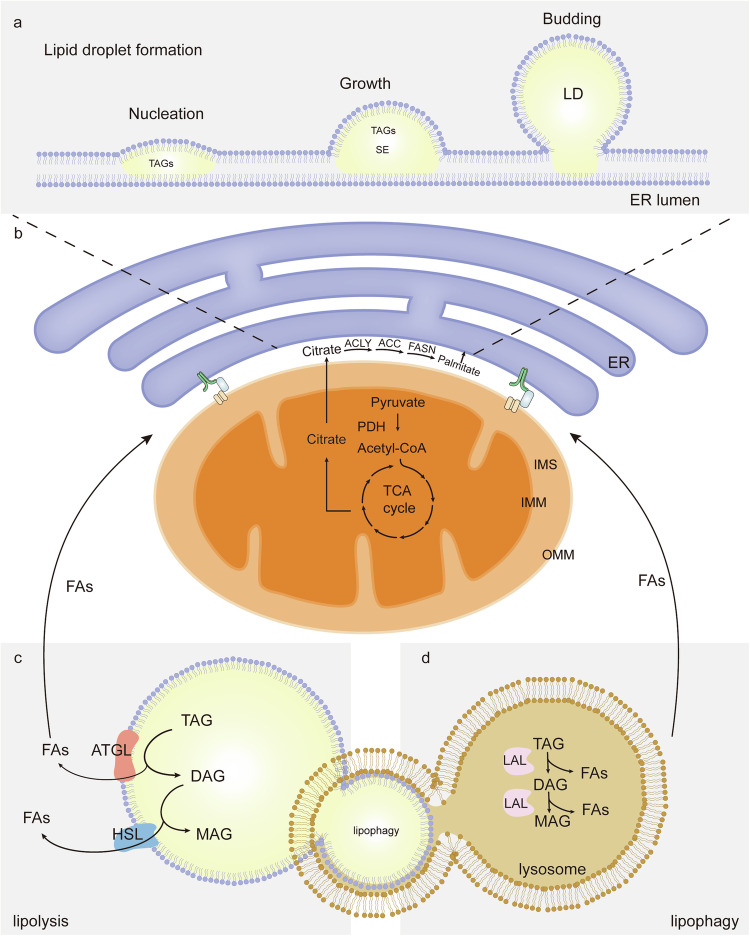
The formation of LDs and canonical pathway of lipolysis. **a** The biogenesis of LDs can be categorized into three primary stages: nucleation, growth and budding. **b** The conversion of citrate into palmitate occurs consecutively in the intermembrane space between the ER and the OMM, facilitated by ACLY, ACC and FASN enzymes. Subsequently, palmitate permeates into the two leaflets of the ER membrane to produce TAG via enzymatic action. **c** The process of lipolysis involves sequential hydrolysis of TAG and DAG by ATGL and HSL, resulting in the production of free FAs. **d** The process of lipolysis involves the segregation of FAs from large LDs through lipolysis, followed by hydrolysis of small LDs by lipophagy to generate free FAs.

The flow of carbons in FAs originates from nutrients like glutamine or glucose, which undergoes a sophisticated cascade of enzymatic reactions [19]. The cytoplasmic acetyl-CoA, serving as the metabolic intermediate that furnishes the substrate for FAs, can be derived either from citrate. Acetyl-CoA reacts in the mitochondria to produce citric acid, which is then transported to the cytosol [16]. The DNL pathway commences with the conversion of citrate into acetyl-CoA, catalyzed by ATP-citrate lyase (ACLY) [22]. The resulting acetyl-CoA serves as the substrate for acetyl-CoA carboxylases (ACC) to undergo carboxylation, thereby giving rise to the formation of malonyl-CoA. The pivotal role of this step in DNL lies in its representation as a key rate-limiting process, attributed to the irreversible conversion of acetyl-CoA into malonyl-CoA [17]. ACC is regulated by phosphorylation, which is essential for the activity of malonyl-CoA determines carnitine palmitoyltransferases 1 (CPT1). Fatty acid synthase (FASN) represents the final and most complex step in the synthesis of palmitate, culminating in the production of the exquisitely saturated 16-carbon FA palmitate (Fig. 1b) [22]. The elongated and desaturated of palmitate in the ER can be facilitated by the enzymatic activities of stearoyl-CoA desaturase-1 (SCD), glycerol-3-phosphate acyltransferase (GPAT) and acylglycerolphosphate acyltransferase (AGPAT2), resulting in the generation of a plethora of FA species including phosphatidic acid (PA) [19]. PA, an important class of structural and signaling phospholipids, is a key substrate for synthesizing diacylglycerols (DAG), which can be used to produce TAG [23].

The intracellular LDs in adipocytes are composed of a hydrophobic core containing TAG and sterol esters (SE), which are enveloped by a phospholipid monolayer associated with LDs proteins [24]. The formation of LDs originates from the ER and can be classified into three distinct stages: nucleation, growth and budding (Fig. 1a). The nucleation step is the accumulation of newly synthesized TAG and SE amidst the interplay of two leaflets of the ER membrane, giving rise to an oil lens structure. The growth phase is distinguished by the process of maturation, where molecules gradually diffuse from a smaller volume of LDs to a larger volume of LDs. Finally, the budding step is typically accomplished in mammalian cells. Following the budding process, LDs can still transfer TAG from the ER to LDs through the process of LD-LD fusion. The majority of cells generate LDs within a size ranging from 0.1 to 10 μm [25].

Although the composition and formation process of intracellular LDs is same in tumor cells and other cells, tumor cells need to increase DNL, FAs uptake and FAO for energy production and lipid accumulation. Therefore, the DNL process in the intermembrane space between ER and OMM is over-activated in tumor cells than that of normal cells, while the formation of LDs and substances exchange in two leaflets of the ER membrane are accelerated in tumor cells accordingly [18]. The lipids synthesized through DNL or derived from exogenous sources accumulate within the ER, ultimately facilitating the formation of LDs. Once stored in LDs, TAG is converted to free FAs through lipolysis and lipophagy. These FAs are then degraded through mitochondrial β-oxidation, which provides the energy required by rapidly proliferating cells [26].

### The process of lipolysis and lipophagy in tumor microenvironment

Tumor cells utilize LDs as temporary storage for FAs, which serve as a reservoir during the dynamic conditions that characterize the tumor microenvironment, encompassing hypoxia or reoxygenation as well as periods of feeding or starvation [27]. LDs function as regulatory switches that coordinate the trafficking and utilization of FAs for energy generation, maintenance of redox homeostasis, or membrane biogenesis amidst phases of rapid cell proliferation. Lipolysis and lipophagy represent two pivotal processes implicated in the breakdown of TAG stored within LDs to subsequently fuel oxidation within mitochondria [28]. Many studies have demonstrated that there is a significant interdependence between these two processes. Specifically, adipose triglyceride lipase (ATGL), the primary enzyme responsible for lipolysis, predominantly acts on larger LDs (>1μm in diameter), while targeting of lipophagy is restricted to smaller cytoplasmic LDs populations (<1μm in diameter) [29]. The process of lipophagy occurs within lysosomes, which is the mode of degradation of LDs. Here, lysosomal acid lipase (LAL) acts to break down TAG into FAs and glycerol [30, 31]. It can be inferred that both lipolysis and lipophagy directly contribute to LDs catabolism; however, the pathway of lipophagy can be considered a rapid and primary elimination process aimed at generating FAs for subsequent β-oxidation, which cancer cells exploit to sustain continuous growth and proliferate [32].

In the process of lipolysis, cytosolic lipases including ATGL, hormone-sensitive lipase (HSL) and monoglyceride lipase (MGL), work in sequence to free the three FAs units that make up the original TAG molecule [33]. The process of lipolysis is believed to involve the catabolism of TAG that are associated with large LDs. Patatin-like phospholipase domain containing 2 (PNPLA2), also known as ATGL, belongs to the PNPLA family, which comprises nine patatin-like phospholipase domain-containing proteins(PNPLA1-9), all of which contain a conserved N-terminal protein domain [34]. ATGL catalyzes the hydrolysis of TAG to form DAG and free FAs (Fig. 1c). The ATGL stands out as the only potent TAG hydrolase within this family, preferentially cleaving the ester bond at Sn1 or Sn2 position. HSL completes this process by hydrolyzing diacylglycerols into monoacylglycerols and free FAs. HSL has broader substrate specificity than ATGL, as it predominantly hydrolyzes FAs residues located at the sn-1 or sn-3 position of diacylglycerols. The final step involves the MGL enzyme’s stereospecific cleavage of monoacylglycerols derived from both triacylglycerols and glycerophospholipids in all three positions. The presence of MGL can be observed in the cytoplasm, unlike ATGL or HSL, it does not exert its action on LDs [17, 35]. The substrate specificity of ATGL for TAG is magnified by tenfold compared to DAG, thereby selectively initiating the initial step of TAG hydrolysis and resulting in the generation of DAG and FAs [36]. The large LDs are perhaps preferentially degraded by lipolysis via ATGL, the linchpin enzyme in the process [37].

The degradation of small LDs in lysosomes via autophagic processes gives rise to the generation of metabolite. The process of lipophagy involves the hydrolysis of lipids that are transported into lysosomes via a receptor-mediated pathway called lipoprotein endocytosis. Lipophagy is orchestrated by lipases including LAL and occurs at an acidic pH within the lysosome. LAL hydrolyzes TAG and DAG, while it is controversial whether it can hydrolyze MAG (Fig. 1d) [35]. In this context, lipophagy is an appealing mechanism for the rapid elimination large amounts of cellular lipid [38]. Although lipolysis and lipophagy exhibit distinct morphological and mechanistic characteristic, there is an interaction between the two pathways [39]. The ATGL enzyme catalyzes the breakdown of large LDs (>1μm), producing small LDs (<1μm) more suitable for the subsequent LAL-mediated lipophagy process [31].

Collectively, it is widely acknowledged that cancer cells exhibit an upregulation of DNL within the intermembrane space bridging the ER and the OMM, resulting in the accumulation of TAG between the ER membrane leaflets and formation of LDs. Upon tumor starvation stress, stored TAG is hydrolyzed into free FAs through lipolysis and lipophagy, which are subsequently transported into mitochondria for further catabolism. The aforementioned processes are dependent on the presence of the organelles ER and mitochondria. Moreover, there is ample evidence to support the idea that collaborative functional loop between the membranous network of ER and mitochondria plays a crucial role in facilitating signaling transduction, protein clusters function, FAs and Ca^2+^ transport, as well as regulation in respiratory chain during lipid metabolism, ultimately influencing cancer cells development.

## The ERMMDs

Previously, names as mitochondria-associated membranes (MAMs), mitochondria-ER contacts (MERCs), ER-Mitochondria contact sites (ERMCS) and ER-mitochondria encounter structure (ERMES) are defined to state the contact sites/membranes where the two organelles are closely apposed but the membranes do not fuse, thus the organelles each maintain their identities [40–42]. In the present review, alterations in the lipid metabolism of tumor cells are intricately intertwined with the bioenergetics functions spanning a more expensive membranous network of ER and mitochondria, newly defined as ERMMDs, affording the collaborative functional loop of these two organelles for abnormal lipid metabolism. Then, the structure of ERMMDs and their functions as substance transportation, signaling transmission and coordination are discussed as following.

### The structure of ERMMDs

**Fig. 2:**
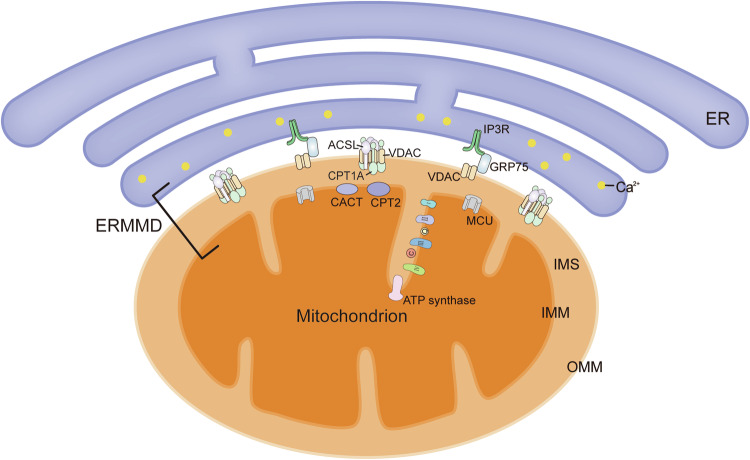
The structure of ERMMDs. The ERMMDs consist of ER membranous system as well as the IMM, OMM, and the intermembrane space, on which a variety of proteins include CPTIA-VDAC-ACSL clusters, IP3R-GRP75-VDAC protein, CACT, CPT2, complex I to IV and ATP synthase, CoQ10, and cytochrome c, etc.

The ER tubular network establishes numerous contacts with other organelles, including with mitochondria to form the ERMMDs. The ERMMDs constitute the extensive ER membranous system as well as the physical contacts of ER-mitochondrial interactive network including the IMM, the OMM, and gap between the membranes (Fig. 2). There are thousands of proteins present on the ERMMDs. For instance, there exists a specific and functional protein cluster known as CPT1A-VDAC-ACSL located in the OMM, which plays a pivotal role in facilitating lipid transfer to mitochondria. Moreover, gap distance between ER-OMM is 6–15 nm, such a short tethering distance between which allows for the translocation of precursor substances, ER-mitochondrial functional collaboration and signal transduction.

The mitochondria serve as bioenergetic, biosynthetic and signaling organelles, enabling them to function as crucial cellular stress sensors that facilitate cellular adaptation to the environment. Furthermore, they confer flexibility for the growth and survival of tumor cells, thus playing a pivotal role in tumorigenesis [43]. The IMM is highly invaginated, forming cristae that provide an expanded surface area for the oxidative phosphorylation system. The IMM has oxidative phosphorylation system comprising the respiratory complex I to IV and ATP synthase, and membrane proteins such as acylcarnitine translocase (CACT), the mitochondrial Ca^2+^ uniporter (MCU) and carnitine palmitoyltransferase (CPT2) [44]. The OMM is covered with a number of membrane proteins, such as long chain acyl-CoA synthetase (ACSL), carnitine palmitoyltransferase 1 A (CPT1A) and the voltage-dependent anion channel (VDAC). The experimental evidence unequivocally demonstrates a robust physical interplay among the OMM proteins CPT1A, ACSL and VDAC, culminating in formation of exquisite CPT1A-VDAC-ACSL clusters [45]. The basic principle behind the mitochondrial energy factory designation is the generation of cellular energy through OXPHOS. The TCA cycle produces NADH and FADH_2_, which serve as crucial inputs for the OXPHOS system [46].

The ER, the most extensive organelle in cells, is responsible for protein synthesis, lipid and steroid production, and Ca^2+^ storage. The ER is made up of the nuclear membrane and the peripheral endoplasmic reticulum, which consists of sheets and tubules. ER sheets synthesize abundant secreted proteins; while ER tubules mainly participate in lipid synthesis and Ca^2+^ signaling, etc [45]. For example, the synthesis of most proteins and essential lipids within mitochondria is limited, necessitating their importation from external sources. The ER is the primary site for the synthesis of phospholipid, which can subsequently be acquired by mitochondria via the ERMMDs [47]. Besides, the ER stores large amounts of Ca^2+^ within the cells, and Ca^2+^ signaling between the ER and mitochondria is crucial in cancer [48]. The ER communicates with the mitochondria via ERMMDs to regulate various aspects of mitochondrial [49].

The ERMMDs, as the most direct medium of communication between mitochondria and ER, play a key role in the collaborative functional loop between these two organelles. Lipid microdomains, also known as lipid rafts, are detected within ERMMDs that enriched in cholesterol and sphingolipids [50]. The presence of raft-associated lipids within these microdomains directly or indirectly regulates channel activity [51]. Notably, various types of ion channels including Ca^2+^ channels are present within these microdomains and play a crucial role in regulating of cell survival and death processes [52]. Studies have demonstrated that the production of inositol 1,4,5-triphosphate receptors (IP3R) exclusively occurs within lipid microdomains. Furthermore, IP3R, VDAC and MCU are recruited to lipid micridomains where they form functional complexes responsible for mediating Ca^2+^ transfer. Given that Ca^2+^ serves as a critical second messenger controlling diverse cellular processes, including cell survival and death [51].

### The function of ERMMDs

#### Substance transportation

The substance transportation of ERMMDs includes not only FAs serving as energy sources, but also lipids related to membrane structure, such as the precursor substance of CL, PA. FAs serve as a vital reservoir of energy within the mitochondria and undergo oxidation in the matrix through β-oxidation, while they entry into the mitochondria occurs via membrane proteins located on ERMMDs. TAG is hydrolyzed by lipase to produce glycerol and FAs, which enter the mitochondrial IMS through the CPT1A-VDAC-ACSL clusters and subsequently enter the mitochondrial matrix via CACT and CPT2 [8]. The IMM is rich with CL, which is associated with the electron respiratory chain on the IMM [53]. Lipids synthesized by the ER are normally transported to other organelles via vesicles, but rapid transport can also occur via non-vesicular transport mechanisms, e.g. PA translocation at the ERMMDs. Then, PA undergoes a series of enzymatic reactions to produce CL [54].

In addition to some substances of precursors that are transported into mitochondria via ERMMDs, there is also some transport into the ER. DNL culminates in the production of the saturated 16-carbon FA palmitate. Palmitate enters the ER and undergoes a captivating series of enzymatic reactions, ultimately giving rise to exquisite neutral lipids like sterol esters SE or TAG. Subsequently, TAG accumulation subsequently takes place within the ER bilayer, leading to the formation and buildup of neutral lipid lenses. The ER bilayer forms droplets in the cytoplasm to create LDs when enough TAG accumulates [55].

#### Signaling transmission

The ER is the main intracellular storage site for Ca^2+^. Calcium channels are present on the ERMMDs, and Ca^2+^ are released from the ER via IP3R, taken up by VDAC on the OMM, and then translocated to the mitochondrial matrix by the MCU on the IMM [45]. After entering the mitochondrial matrix, Ca^2+^ can bind directly to isocitrate dehydrogenase (ICDH) and α-ketoglutarate dehydrogenase (KDH). Activation of Ca^2+^ triggers these enzymes, thereby increasing electrons flow through the respiratory chain and boosting ATP production [45]. The regulation of Ca^2+^ signaling is crucial for cellular bioenergetics because enzymes in the TCA cycle and the electron transport chain (ETC) rely on Ca^2+^ to produce ATP, an energetically rich molecule.

#### Functional coordination

The coordination between mitochondria and ER regulates intracellular lipid metabolism via ERMMDs. The entry of Ca^2+^ into mitochondrial matrix via ERMMDs alters mitochondrial activity in various ways. For example, Ca^2+^ can activate dehydrogenases in the TCA, or activate ATP synthase. In addition, CL is produced in the IMM and plays a crucial role in binding substance for cytochrome c in the respiratory chain [53].

## CPT1A-VDAC-ACSL clusters on ERMMDs participate in FAs transport

The OMM of ERMMDs hosts a collective assembly of CPT1A, ACSL and VDAC, forming exquisite CPT1A-VDAC-ACSL clusters through robust physical interactions that are crucial for the rate-limiting step of FAs transportation into the mitochondria. Then, how FA enters mitochondria from ER-derived LDs through ERMMDs, as well as the regulation of CPT1A by malonyl-CoA, a metabolite of ACC, forming the AMPK-ACC-CPT1A axis will also be described in detail.

### The structure of CPT1A-VDAC-ACSL clusters

The formation of a cluster involving CPT1A, ACSL and VDAC, is observed in the ERMMDs. The OMM proteins CPT1A, ACSL and VDAC have been shown to engage in robust physical interactions. The proteins, CPT1A, ACSL and VDAC, possess strong hydrophobic characteristics within their membrane structures. The formation of a stable complex relies on the intricate hydrophobic interactions between these individual proteins [8].

In mammals, CPT1 exists three distinct isoforms, encoded by separate genes. The positioning of CPT1A and CPT1b within the OMM acts as a crucial portal, enabling the influx of FAs into mitochondria for subsequent oxidative processes. CPT1c, the final member of the CPT1 gene family, is found in the ER.

CPT1A, with a size of approximately 88 kDa, represents the predominant hepatic isoform. However, it is also present in various other tissues such as heart, spleen, adipose tissues and kidney. CPT1b, with a size of approximately 88 kDa, is expressed in adipose tissue, testis and cardiac muscle [56]. The expression of CPT1A and CPT1b exhibits species- and gender-specific disparities. For example, the expression patterns of CPT1A and CPT1b undergo developmental changes, wherein the level of cardiac CPT1A expression are initially elevated around birth but subsequently decline, while CPT1b exhibits an inverse trend [57]. Expression of CPT1b in the liver is induced under specific circumstances [58].

The catalytic efficiency of CPT1c is relatively low when utilizing carnitine and acyl-CoA as substrates, despite possessing all the necessary components for enzymatic activity [59]. A recent discovery has revealed an intriguing role for CPT1c in lipid metabolism, specifically its involvement in the synthesis of LDs [60]. CPT1c promotes acyl-CoA accumulation in TAG rather than their oxidation in mitochondrial, making the subcellular localization of CPT1c a relevant factor. CPT1c indirectly affects TAG metabolism by affecting the expression of TAG metabolism in ER membranes [59].

VDAC, also termed mitochondrial pore protein, is positioned within the OMM and plays a vital role in orchestrating mitochondrial-mediated apoptosis [61]. In addition to governing mitochondrial metabolism and energy function, VDAC is also involved in maintaining Ca^2+^ homeostasis, safeguarding against oxidative stress and apoptosis regulation [62]. The identification of three distinct subtypes of VDAC, namely VDAC1, VDAC2 and VDAC3, has been accomplished. VDAC1 was the most abundant of the three isoforms [63]. PA22 is a 22-kDa polyanionic VDAC inhibitor that is selective for the first two steps of FAO and does not affect other substrates [8].

### CPT1A-VDAC-ACSL clusters facilitate the transport of FAs to mitochondria

**Fig. 3:**
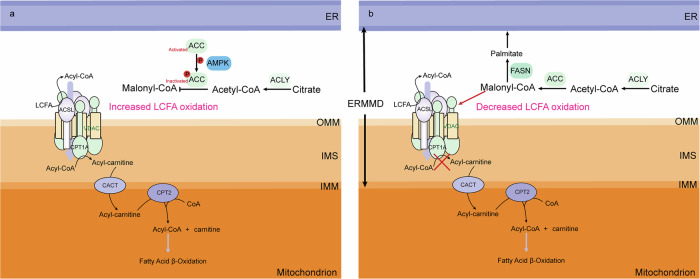
The regulation of ACC and CPT1A by AMPK controlled lipid transport through CPT1A-VDAC-ACSL clusters. **a** The activation of AMPK leads to the inhibitory phosphorylation of ACC, reducing the expression of malonyl-CoA and thereby releasing the inhibition on CPT1A. This results in an increased entry of long-chain fatty acid (LCFA) into the mitochondria for β-oxidation. **b** ACC is an important enzyme in FAs synthesis, catalyzing the carboxylation of acetyl-CoA to form malonyl-CoA. The catalysis of malonyl-CoA by FASN results in the production of palmitate while simultaneously inhibiting CPT1A.

The function of FAs in the energy production of tumor cells is crucial, as they are shuttled into mitochondria through the carnitine transport mechanism. The carnitine shuttle encompasses the intricate interplay of CPT1A-VDAC-ACSL clusters, CACT and CPT2, facilitating the translocation of FAs into the mitochondrial matrix through an ester exchange reaction, subsequently paving the way for their β-oxidation. FAs are activated by ACSL in the CPT1A-VDAC-ACSL clusters, and CPT1A converts activated FAs to acyl-carnitine, which are then translocated to the IMS via VDAC. Subsequently, CACT further transfers acyl-carnitine into the mitochondrial matrix, where it undergoes conversion to acyl-CoA in the presence of CPT2, followed by FAs β-oxidation (Fig. 3a) [64].

### AMPK-ACC-CPT1 axis regulates mitochondrial FAs transport

The AMPK-ACC-CPT1 axis regulates the oxidation of mitochondrial FAs, including short, medium and long chain FAs [65]. However, since medium and FAs are not free to enter mitochondria across the membrane, they need to pass through CPT1A on the mitochondrial membrane. The study establishes a direct correlation between FAs oxidation efficiency and CPT1A activity [66]. The first precursor of DNL is Malonyl-CoA, which also serves as inhibitor of CPT1A [67]. Activated AMP-activated protein kinase (AMPK) activity can deactivate ACC via phosphorylation, while ACC is tightly regulated through phosphorylation and metabolic control. The regulation is crucial as the indispensable role played by malonyl-CoA in determining the activity of CPT1A, which facilitates the coupling of acyl chains with carnitine for efficient translocation into the mitochondrial matrix for subsequent degradation through β-oxidation (Fig. 3) [66].

AMPK contains three subunits: a 63 kDa α subunit, a 38 kDa β subunit and a 38 kDa γ subunit. The α subunit serves as the catalytic component while the β and γ subunits function as regulatory subunits [68]. The involvement of AMPK extends to various facets of cellular and organismal lipid metabolism, encompassing the oxidative catabolism of FAs and TAGs, regulation of cellular metabolic processes and promotion of cell proliferation [69]. The capacity of AMPK to regulate lipid metabolism is achieved by increasing FAO and autophagy, while reducing cholesterol and FAs synthesis [70].

The ACC enzyme is crucial for regulating lipogenic reactions and mitochondrial β-oxidation. There are two subtypes of ACC, namely ACC1 and ACC2. ACC1, approximately 265-kDa in size, is a protein predominantly expressed in adipose tissue and liver [71]. ACC2, approximately 280-kDa in size, is a mainly expressed in muscle, liver and several other organs [56]. The evidence suggests that malonyl-CoA produced by ACC serves not only as a facilitator of lipogenesis but also crucially regulates CPT1A activity.

The natural inhibitor of CPT1 is Malonyl-CoA, which is catalyzed by ACC. CPT1A and CPT1b have different sensitivities to malonyl-CoA, with the latter being 100 times more sensitive than the former [56]. The malonyl-CoA produced by ACC is mainly used for DNL and functions as an inhibitor of CPT1 [71].

## Mitochondrial energy metabolism pathways regulated by collaborative loop of ERMMDs

Increasing ERMMDs mediated collaborative loop may be an adaptive mechanism driven by tumor starvation stresses, aiming to enhance mitochondrial functionality and facilitate lipid synthesis for energy supplying. During cancer cell starvation stress, FAs are ushered into the mitochondria via ERMMDs for β-oxidation and subsequent integration into the TCA cycle. This process involves a series of oxidation reactions that generate NADH and FADH_2,_ which serve as electrons reservoirs [53]. These carriers assist in transferring electrons to the mitochondrial oxidative respiratory chain embedded in the IMM, which in turn uses this process to drive protons into the IMS [72, 73]. Simultaneously, protons traverse an electrochemical gradient via ATP synthase to generate ATP, which serves as the primary energy carrier in nearly all cellular processes [74]. The mitochondrial oxidative respiratory chain is a crucial component of cellular metabolism, consisting of five enzyme complexes and two electron carriers. The ER can regulate the aforementioned processes via ERMMDs. For instance, Ca^2+^ stored in the ER can trigger the activation of TCA cycle enzymes, thereby regulating metabolic speed. Furthermore, the electron carrier cytochrome c in the respiratory chain needs to bind to CL, whose synthesis requires its precursor lipids of PA imported from ER to mitochondrial membranes and occurs at the IMM [18]. These collaborative loops worked in multiple membranous networks of ERMMDs will be described in more detail below.

### FAO

There are multiple pathways for FAO, including mitochondrial β-oxidation, α-oxidation and ω-oxidation within the ER. The degradation of acyl-CoA occurs within the mitochondria through a cyclic process called β-oxidation, which involves four enzymatic steps [75]. The generation of acetyl-CoA is achieved in each cycle by releasing two carbon atoms from the carboxyl-terminal of acyl-CoA. The cycle commences with the transformation of acyl-CoA into trans-2-enoyl-CoA via the catalytic action of acyl-CoA dehydrogenase. This is followed by a hydration step catalyzed by enoyl-CoA hydratase, which produces (S)-3-hydroxyacyl-CoA. The compound is dehydrogenated by (S)-3-hydroxyacyl-CoA, facilitated by 3-ketolipoyl-CoA dehydrogenase. The thiolase enzyme finally breaks down 3-ketolipoyl-CoA into acetyl-CoA with a shortened double-carbon chain acyl-CoA. The β-oxidation cycle of each FA reduces its length by two carbon atoms, while producing acetyl-CoA, FADH_2_, and NADH [76].

Each enzyme is also sensitive to feedback inhibition of the products of enzymatic reactions, including FADH_2_ and NADH. Under conditions of low metabolic activity, reduced activity in both the ETC and TCA cycle results in an accumulation of acetyl-CoA, FADH_2_ and NADH, which in turn feeds back to inhibit FAs β-oxidation.

### The transmission of Ca^2+^ in TCA cycle

**Fig. 4:**
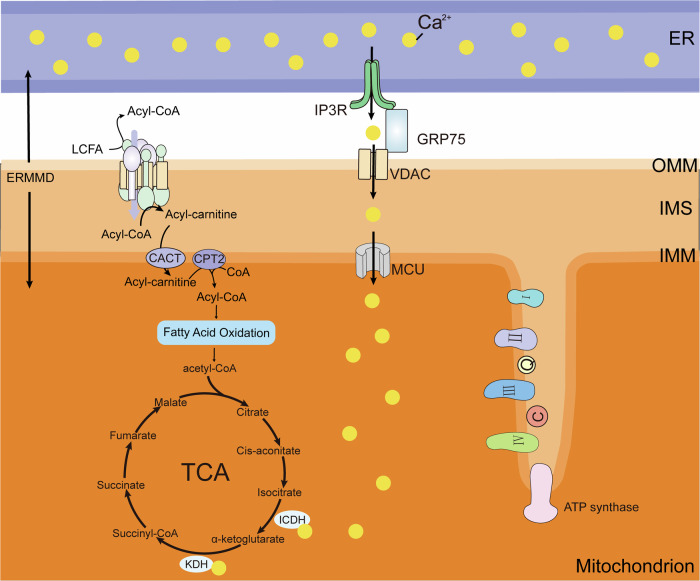
The role of Ca^2+^ plays a crucial role in the regulation of biological energy. Cancer cells rely on pro-survival Ca^2+^ transmission from ER to mitochondria via ERMMDs and enhance mitochondrial metabolism through Ca^2+^-dependent TCA stimulation. Ca^2+^ enters the IMS through IP3R-GRP75-VDAC on ERMMDs and subsequently penetrates the mitochondrial matrix via MCU. Increased Ca^2+^ concentration activates ICDH and KDH, thereby increasing electron flux through the respiratory chain and ATP production.

Increasing transmission of Ca^2+^ into mitochondrial matrix required by cancer cells contributes to their subsequent activation of dehydrogenases in TCA cycle for more energy production to meet the cells rapid proliferation. The presence of Ca^2+^ is indispensable for regulating mitochondrial bioenergetics and metabolism, serving as a vital cofactor for various rate-limiting TCA enzymes such as ICDH and KDH [77]. The maintenance of cellular bioenergetics relies on transfer of Ca^2+^ from ER to the mitochondria via the ERMMDs [78]. The primary conduit for Ca^2+^ into mitochondria is throughout IP3R-GRP75-VDAC1 complex located on the ERMMDs [79]. Subsequently, Ca^2+^ traverses the IMM via the MCU complex, which stands as the sole known pathway for Ca^2+^ to access the mitochondrial matrix [80]. Ca^2+^ entering the mitochondria appear to influence the activity of certain enzymes, including mitochondrial ATP synthase, ICDH and KDH [78]. Ca^2+^ stimulates dehydrogenase, leading to increased energy production in cells. ICDH and KDH bind Ca^2+^ directly, leading to changes in the kinetics of substrates and inhibitory metabolites (Fig. 4) [81].

Mitochondrial Ca^2+^ addiction is a characteristic of cancer cells; in other words, cancer cells maintain their tumorigenic vitality by transporting Ca^2+^ through ERMMDs [9, 82]. For example, studies have found that when the absorption of Ca^2+^ in mitochondria is blocked or the release of Ca^2+^ facilitated by IP3R in ERMMDs is inhibited, tumor cells will undergo a bioenergetic crisis similar to normal cells [83]. Raturi et al. demonstrated that ER-localized thioredoxin-related transmembrane protein 1(TMX1) targeting ERMMDs in a palmitoylation-dependent manner enhances mitochondrial ATP production and induces apoptosis in tumor cells by modulating the number of ER-mitochondria contacts and intracellular ER-mitochondria Ca^2+^ flux, thereby inhibiting tumor growth [84]. Research finding have underscored the critical role of Ca^2+^ exchange through the ERMMDs in mitochondrial energy production and the determination of cellular outcomes in tumor cells.

Acetyl-CoA can be obtained from a variety of sources, including carbohydrates. Glucose is transformed into pyruvate by the process of glycolysis, which is then decarboxylated to yield acetyl-CoA. β-oxidation of FAs produces acetyl-CoA, which is the begin of TCA cycle. The conversion of acetyl-CoA and oxaloacetate to citrate is catalyzed by the enzyme citrate synthase. The conversion of citrate to cis-aconitate is catalyzed by aconitase. Cis-aconitate serves as an intermediate that can be further transformed into isocitrate through the action of aconitase. Isocitrate dehydrogenase converts isocitrate to α-ketoglutarate. In addition, this step also produces NADH cofactor. The α-ketoglutarate dehydrogenase catalyzes the conversion of α-ketoglutarate to succinyl-CoA. Succinyl-CoA is subsequently converted to succinate by the succinate by the enzymatic of succinate thiokinase. The conversion of guanosine diphosphate (GDP) to guanosine triphosphate (GTP) occurs in this reaction. Succinate dehydrogenase catalyzes the transformation of succinate into fumaric acid. In this reaction, FAD is transformed into FADH_2_. Fumaric acid is produced as malate by the action of Fumarate Hydratase. The process of malate dehydrogenation, catalyzed by malate dehydrogenase, leads to the production of oxaloacetate. This reaction also leads to the conversion of NAD to NADH, while the oxaloacetate generated from this process engages with acetyl-CoA to give rise to citrate, thereby initiating another cycle of the illustrious citric acid pathway [85]. The NADH and FADH_2_ produced during this process will then enter the oxidative respiratory chain for further reactions to produce ATP.

### CL affect OXPHOS

**Fig. 5:**
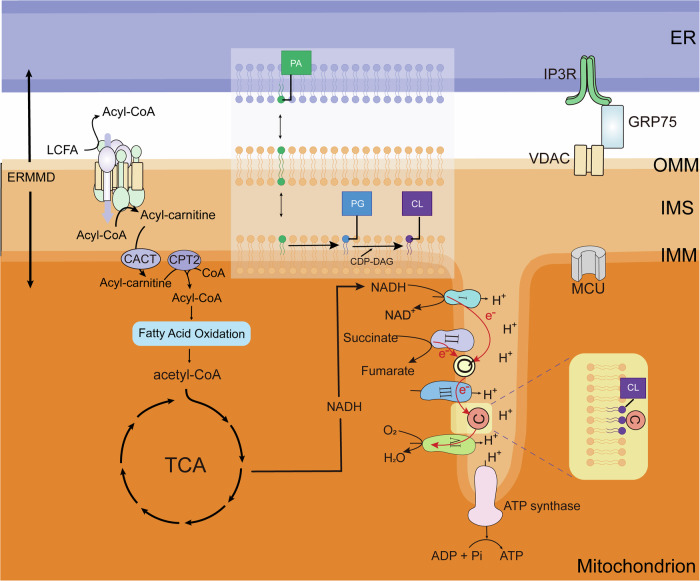
Lipid transport at ERMMDs and mitochondrial respiratory chain complex. Lipids and their precursors are synthesized in the ER and transported into mitochondria via ERMMDs. CL synthesis occurs at the IMM and requires precursor lipids PA from the ER at ERMMDs. The presence of CL is crucial for the optimal functioning of cytochrome c within the respiratory chain located on the IMM. The mitochondrial respiratory chain, which consists of four complexes and two coenzymes, enables ATP production through OXPHOS. The red arrows indicate electron shuttle in the respiratory chain. Complexes I, III and IV are proton pumps that allow protons to pass from the matrix to the IMS. Once the IMS is enriched with protons, ATP synthase allows them to pass in the direction of the gradient while producing ATP.

There are many enzymes involved in phospholipid biosynthesis in the ERMMDs, which plays a pivotal role in phoshpolipid exchange between the ER and mitochondria [53]. The unique conical molecular structure of CL primarily located in the IMM, enables them to regulate mitochondrial respiration through their binding affinity with cytochrome c [86]. The interplay between CL and cytochrome c is crucial for determining the functionality of the IMM, while cytochrome c also plays a key role in transferring electrons from complex III to complex IV within the ETC. Consequently, alterations in lipid composition within cancer cells can directly impact cellular bioenergetics by modulating ETC activity [87]. The majority of CL is synthesized from phosphatidic acid (PA) derived from ER through the enzyme cascade on the matrix side of the IMM, and subsequently redistributed between both membranes (Fig. 5) [88].

The mitochondrial OXPHOS system is essential for cellular energy metabolism and ATP production in many cancers [7]. Five complexes and two mobile electron carriers make up the respiratory chain. These complexes are identified as complex I (NADH-ubiquinone oxidoreductase), Complex II (succinate dehydrogenase), Complex III (ubiquinol-cytochrome c oxidoreductase), Complex IV (cytochrome c oxidase) and ATP synthase. The two mobile electron carriers encompass ubiquinone and cytochrome c (Fig. 5). The compound ubiquinone, also referred as coenzyme Q10 (CoQ10), diffuses within the IMM and donates electrons to complex III. The cytochrome c is positioned on the intermembrane space side of the IMM, where it bestows its electrons upon complex IV [74].

The Complex I consists of a total of 44 distinct subunits, with seven encoded by mtDNA and the rest derived from nuclear DNA [89, 90]. Complex I serve as the primary gateway for electrons to embark on their journey through the ETC, orchestrating the transfer of a pair of electrons from NADH oxidation to the electron carrier ubiquinone [91, 92].

The sole constituent that fails to facilitate proton pumping across the mitochondrial membrane is Complex II. The nuclear genome encodes all four subunits of complex II. Complex II has a dual role within the mitochondria: (i) in the TCA cycle, metabolizing succinate to fumarate, and (ii) in the ETC, facilitating the transfer of electrons from the reduction of flavin adenine nucleotides to the electron carrier panthenol [93, 94].

Complex III has a total of 11 subunits. It orchestrates the coupling between ubiquinol oxidation and cytochrome c reduction, while simultaneously acquiring two protons from the matrix and liberating four protons into the IMS. Complex III has a functionally relevant homodimeric structure with three catalytic subunits per monomer: cytochrome b, cytochrome c1 and rieske iron-sulfur protein [74].

Cytochrome IV is composed of a total of 14 subunits. Three of these subunits are catalytic in nature and encoded by mtDNA, while the rest are encoded in the nuclear genome [95]. The electrons transferred by cytochrome c are accepted by Complex IV, which then reduces oxygen to water [96].

The proton gradient produced by complexes I, III and IV, is utilized by ATP synthase to generate ATP from ADP. ATP synthase possesses two functional domains: the hydrophilic substrate F1 domain, which is responsible for ATP production, and the Fo domain facing the membrane, which facilitates proton translocation [97].

The transfer of two electrons to CoQ10 is carried out by both Complexes I and II. The two electrons transferred from complex I originate from NADH oxidation, while the two electrons transferred from complex II are from the oxidation of succinate to fumarate. The presence of CoQ10 facilitates electron transfer to complex III. Electrons are then transferred from Complex III to cytochrome c, thereby establishing a connection with complex IV. Complex IV reduces O_2_ to H_2_O molecules. The function of Complex V is to facilitate the passage of protons along the gradient and harness their flow to catalyze the conversion of ADP into ATP [98].

The production of Acetyl-CoA can occur through both sugar and fatty acid metabolism, but there is a crucial distinction exists: glycolysis generates two acetyl-CoA molecule substrates for every six carbon atoms and FAs β-oxidation produces three, meaning that the predominant energy source is FAs rather than sugar and the OXPHOS system is more efficient at producing ATP [74]. In order to meet the rapid growth needs of tumors, relying solely on glucose metabolism for energy is not enough; instead, lipid metabolism becomes over-activated. Therefore, the alteration in energy metabolism is considered a hallmark of cancer and also an important target for cancer treatment [99].

## Conclusion and future perspectives

As lipid metabolism is pivotal source of high-energy fuel that is often altered in neoplasia to meet the cells rapid proliferation, ERMMDs mediated abnormal lipid metabolism provides a systematic angle to reveal the collaborative functional loop between mitochondria and ER [100]. We summarized the events occurred in ERMMDs through DNL, FAs stored as LDs and their catabolism, CPT1A-VDAC-ACSL clusters participated FAs transport, as well as ERMMDs mediated collaborative loop of FAO, Ca^2+^ transmission in TCA cycle, and OXPHOS process [2]. The elucidation of these processes will offer a more profound comprehension of the systematic mechanism of lipid metabolism involved in tumorigenesis, which will undoubtedly be instrumental for providing comprehensive strategies for anticancer drugs discovery.

Apoptosis is the most thoroughly examined method of programmed cell death, but recent research has uncovered that there are additional forms of programmed cell death that do not follow the apoptosis pathway [101, 102]. For example, necroptosis and ferroptosis are two distinct forms of regulated necrosis that are controlled by specific genetic mechanisms and may operate in various physiological and pathological scenarios [103]. In 2012, Dixon and his team introduced the concept of ferroptosis, which refers to a type of cell death that is regulated and dependent on iron. This form of cell death is triggered by an excessive buildup of lipid peroxides on the cellular membrane [102]. In terms of cancer cell properties, to meet the energetic and nutrient requirements for survival and rapid proliferation of cancer, enhanced lipid metabolism is executed to generate adenosine triphosphate, and even accumulation of lipid peroxides as by-product, which make cancer cells more vulnerable to ferroptosis-induced cellular membrane disruption [104]. Moreover, the lethal accumulation of lipid peroxidation products is the main characteristic of ferroptosis, wherein the outcome is determined by an intricate interplay between ferroptosis execution and the defense system at a cellular level. Due to its occurrence on the cell membrane, ferroptosis can potentially affect both organelle membranes and plasma membranes in physiological cells and cancerous cells, and vulnerably happens in the latter [105, 106]. So, induction of ferroptosis has been proposed as promising therapeutic strategy, which may trigger the autophagic cell death process of ferroptosis targeting membrane disruption, especially in membranous system of ERMMDs where lipid metabolism takes place [107].

In order to facilitate rapid growth and proliferation, cancer cells synergistically upregulate lipid metabolism within the ERMMDs, thereby ensuring a steady supply of high-energy fuel and signal-transmitting molecules. The complex changes are intricately occurred in the systematic membranous domains spanning the organelles of ER and mitochondria, among which a series of potential therapeutic targets in ERMMDs are collaboratively worked on tumor progression. First, the high-level lipid metabolism of tumor cells may bring excessive accumulation of lipid peroxidation, which may trigger the autophagic cell death process of ferroptosis with membrane disruption, especially in membranous system of ERMMDs where lipid metabolism takes place [108]. Second, CPT1A-VDAC-ACSL clusters modulated by AMPK-ACC-CPT1 axis and related signaling pathways provide promising targets for FAs and Ca^2+^ transportation between ERMMDs [64]. Finally, ERMMDs mediated collaborative signal transduction on OXPHOS processes may provide potential targets on ATP production, bioenergetic and macromolecular anabolic requirements of cancer cells [74].

However, several uncertainties still persist in the study of ERMMDs, including the composition of the ERMMDs machinery, the limited knowledge regarding different type of protein classification on ERMMDs and how to distinguish their respective functions between the two organelles [49]. The unresolved inquiries will serve as crucial avenues for future research and bring a systemic insight into how the interactive function among these multi-membranes to maintain cellular biology and homeostasis.

## Data Availability

All data generated or analyzed during this study are included in this published article.
